# Draft Whole-Genome Sequence of the Biocontrol Agent *Trichoderma harzianum* T6776

**DOI:** 10.1128/genomeA.00647-15

**Published:** 2015-06-11

**Authors:** Riccardo Baroncelli, Giulia Piaggeschi, Lisa Fiorini, Edoardo Bertolini, Antonio Zapparata, Mario Enrico Pè, Sabrina Sarrocco, Giovanni Vannacci

**Affiliations:** a Dipartimento di Scienze Agrarie, Alimentari e Agro-ambientali, Università di Pisa, Pisa, Italy; b Institute of Life Sciences, Scuola Superiore Sant’Anna, Pisa, Italy

## Abstract

*Trichoderma harzianum* T6776 is a promising beneficial isolate whose effects consist of growth promotion, positive response of photosynthetic activity, hormonal signaling, and carbon partitioning in tomato, coupled with biocontrol of plant pathogens. Here, we present the first genome assembly of T6776, providing a useful platform for the scientific community.

## GENOME ANNOUNCEMENT

*Trichoderma* species are well-known beneficial fungi used as biocontrol agents. The capacity to produce antibiotics and enzymes, to induce systemic resistance in plants, and to parasitize phytopathogenic fungi, coupled with a high metabolic versatility and a strong competition ability, make many *Trichoderma* isolates useful microorganisms for commercial biofertilizers and biopesticides (1–3).

*Trichoderma harzianum* strain T6776 was isolated by the authors from soil in Tuscany, Italy, and it has been studied for many years as a bioactive principle in biostimulants and biopesticides.

The first evidence of its beneficial effects in terms of growth promotion and biocontrol activity against fungal plant pathogens of high economic impact was demonstrated on different tomato cultivars and against *Rhizoctonia solani*, *Fusarium oxysporum* f. sp. *lycopersici*, and *F. oxysporum* f. sp. *radicis-lycopersici* (4). The biostimulating effects of *T. harzianum* T6776, and its endophytic ability, have been further demonstrated under different tomato cultivation systems (soil and hydroponic). Recent data suggest the induction of resistance toward *Alternaria solani* by *T. harzianum*. In addition, significant positive responses of photosynthetic activity, hormonal signaling, and carbon partitioning have been obtained after inoculation of tomato with T6776 (5).

These results encouraged us to produce a complete sequence of *T. harzianum* T6776 as an efficient and powerful tool to investigate the molecular mechanisms controlling the interaction between the fungus and the tomato plant.

The genome of *T. harzianum* strain T6776 was sequenced using Illumina mate-paired sequencing technology by the McGill University and Genome, Quebec Innovation Centre (Canada). Mate-paired reads of 250 bp (3.26 Gbp; average coverage 85×) were assembled using Velvet version 1.2.08 (6). The draft nuclear genome of *T. harzianum* consists of 1,573 sequence scaffolds with a total assembly length 39.73 Mbp (*N*_50_ = 68,846 and *N*_90_ = 15,338), a GC content of 48.50%, and a maximum scaffold size of 330,970 bp. The completeness of the assembly was assessed using CEGMA version 2.4 (7), which estimated the genome sequence to be 98.39% complete. The nuclear genome was annotated using the MAKER pipeline (8). Overall, 11,501 protein-coding gene models were predicted. Analysis with WoLF PSORT (9) revealed that 1,412 predicted proteins (12.28% of the proteome) contain a secretion signal peptide. Among those 63 are *Trichoderma*-specific proteins, of which 35 are strain specific as they do not have any sequence similarity to proteins in public databases, based on BLAST searches (E value threshold of 1E^−3^). Such characteristics are typical of fungal effectors, which modulate plant immunity and facilitate colonization (10, 11). A first comparative analysis within *Trichoderma* spp. (12) and model organisms with publically available genomes (*Fusarium* [13], *Neurospora* [14], *Colletotrichum* [10, 15, 16], *Magnaporthe* [17], *Clonostachys* [18], and *Verticllium* [19]) suggested that *T. harzianum* T6776 contains a large number of carbohydrate-active enzymes (CAZy), such as α-l-fucosidase, chitosanases, α-galactosidase, and alginate lyase (three, five, eight, and three genes, respectively). The genome sequence reported here represents a new resource useful for further research into the biology, ecology, and evolution of biocontrol agents.

### Nucleotide sequence accession numbers.

This whole-genome shotgun project has been deposited in GenBank under the accession number JOKZ00000000 (BioProject PRJNA252551). The version described in this paper is JOKZ00000000.1.

## References

[1] Whipps JM, Lumsden RD 2001 Commercial use of fungi as plant disease biological control agents: status and prospects, p 9–22. *In* Butt TM, Jackson C, Magan N (ed), Fungi as biocontrol agents: progress, problems and potential. CAB International, Wallingford, United Kingdom.

[2] Perotto S, Angelini P, Bianciotto V, Bonfante P, Girlanda M, Kull T, Mello A, Pecoraro L, Perini C, Persiani AM, Saitta A, Sarrocco S, Vannacci G, Venanzoni R, Venturella G, Selosse MA 2013 Interactions of fungi with other organisms. Plant Biosyst 147:208–218. doi:10.1080/11263504.2012.753136.

[3] Jensen DF, Karlsson M, Sarrocco S, Vannacci G Biological control using microorganisms as an alternative to disease resistance. *In* Collinge DB (ed), Biotechnology for plant disease control, in press Wiley, New York, NY.

[4] Sarrocco S, Moncini L, Pachetti G, Moretti A, Ritieni A, Vannacci G 2013 Biological control of *Fusarium* head blight under field conditions. IOCB-WPRS Bull 86:95–100.

[5] Fiorini L, Guglielminetti L, Mariotti L, Curadi M, Puntoni G, Scartazza A, Picciarelli P, Sarrocco S, Vannacci G 2014 *Trichoderma harzianum* 6776 on tomato: a single beneficial isolate for two cultivation systems. J Plant Pathol 95(S4):61.

[6] Zerbino DR, Birney E 2008 Velvet: algorithms for *de novo* short read assembly using de Bruijn graphs. Genome Res 18:821–829. doi:10.1101/gr.074492.107. 18349386 PMC2336801

[7] Parra G, Bradnam K, Korf I 2007 CEGMA: a pipeline to accurately annotate core genes in eukaryotic genomes. Bioinformatics 23:1061–1067. doi:10.1093/bioinformatics/btm071. 17332020

[8] Cantarel BL, Korf I, Robb SM, Parra G, Ross E, Moore B, Holt C, Sánchez Alvarado A, Yandell M 2008 MAKER: an easy-to-use annotation pipeline designed for emerging model organism genomes. Genome Res 18:188–196. doi:10.1101/gr.6743907. 18025269 PMC2134774

[9] Horton P, Park KJ, Obayashi T, Fujita N, Harada H, Adams-Collier CJ, Nakai K 2007 WoLF PSORT: protein localization predictor. Nucleic Acids Res 35:W585–W587. doi:10.1093/nar/gkm259. 17517783 PMC1933216

[10] O’Connell RJ, Thon MR, Hacquard S, Amyotte SG, Kleemann J, Torres MF, Damm U, Buiate EA, Epstein L, Alkan N, Altmüller J, Alvarado-Balderrama L, Bauser CA, Becker C, Birren BW, Chen Z, Choi J, Crouch JA, Duvick JP, Farman MA, Gan P, Heiman D, Henrissat B, Howard RJ, Kabbage M, Koch C, Kracher B, Kubo Y, Law AD, Lebrun MH, Lee YH, Miyara I, Moore N, Neumann U, Nordström K, Panaccione DG, Panstruga R, Place M, Proctor RH, Prusky D, Rech G, Reinhardt R, Rollins JA, Rounsley S, Schardl CL, Schwartz DC, Shenoy N, Shirasu K, Sikhakolli UR, Stüber K, Sukno SA, Sweigard JA, Takano Y, Takahara H, Trail F, van der Does HC, Voll LM, Will I, Young S, Zeng Q, Zhang J, Zhou S, Dickman MB, Schulze-Lefert P, Ver Loren van Themaat E, Ma LJ, Vaillancourt LJ 2012 Lifestyle transitions in plant pathogenic *Colletotrichum* fungi deciphered by genome and transcriptome analyses. Nat Genet 44:1060–1065. doi:10.1038/ng.2372. 22885923 PMC9754331

[11] Spanu PD, Abbott JC, Amselem J, Burgis TA, Soanes DM, Stüber K, Ver Loren van Themaat E, Brown JK, Butcher SA, Gurr SJ, Lebrun MH, Ridout CJ, Schulze-Lefert P, Talbot NJ, Ahmadinejad N, Ametz C, Barton GR, Benjdia M, Bidzinski P, Bindschedler LV, Both M, Brewer MT, Cadle-Davidson L, Cadle-Davidson MM, Collemare J, Cramer R, Frenkel O, Godfrey D, Harriman J, Hoede C, King BC, Klages S, Kleemann J, Knoll D, Koti PS, Kreplak J, López-Ruiz FJ, Lu X, Maekawa T, Mahanil S, Micali C, Milgroom MG, Montana G, Noir S, O’Connell RJ, Oberhaensli S, Parlange F, Pedersen C, Quesneville H, Reinhardt R, Rott M, Sacristán S, Schmidt SM, Schön M, Skamnioti P, Sommer H, Stephens A, Takahara H, Thordal-Christensen H, Vigouroux M, Wessling R, Wicker T, Panstruga R 2010 Genome expansion and gene loss in powdery mildew fungi reveal tradeoffs in extreme parasitism. Science 330:1543–1546. doi:10.1126/science.1194573. 21148392

[12] Kubicek CP, Herrera-Estrella A, Seidl-Seiboth V, Martinez DA, Druzhinina IS, Thon M, Zeilinger S, Casas-Flores S, Horwitz BA, Mukherjee PK, Mukherjee M, Kredics L, Alcaraz LD, Aerts A, Antal Z, Atanasova L, Cervantes-Badillo MG, Challacombe J, Chertkov O, McCluskey K, Coulpier F, Deshpande N, von Döhren H, Ebbole DJ, Esquivel-Naranjo EU, Fekete E, Flipphi M, Glaser F, Gómez-Rodríguez EY, Gruber S, Han C, Henrissat B, Hermosa R, Hernández-Oñate M, Karaffa L, Kosti I, Le Crom S, Lindquist E, Lucas S, Lübeck M, Lübeck PS, Margeot A, Metz B, Misra M, Nevalainen H, Omann M, Packer N, Perrone G, Uresti-Rivera EE, Salamov A, Schmoll M, Seiboth B, Shapiro H, Sukno S, Tamayo-Ramos JA, Tisch D, Wiest A, Wilkinson HH, Zhang M, Coutinho PM, Kenerley CM, Monte E, Baker SE, Grigoriev IV 2011 Comparative genome sequence analysis underscores mycoparasitism as the ancestral life style of *Trichoderma*. Genome Biol 12:R40. doi:10.1186/gb-2011-12-4-r40. 21501500 PMC3218866

[13] Ma LJ, van der Does HC, Borkovich KA, Coleman JJ, Daboussi MJ, Di Pietro A, Dufresne M, Freitag M, Grabherr M, Henrissat B, Houterman PM, Kang S, Shim WB, Woloshuk C, Xie X, Xu JR, Antoniw J, Baker SE, Bluhm BH, Breakspear A, Brown DW, Butchko RA, Chapman S, Coulson R, Coutinho PM, Danchin EG, Diener A, Gale LR, Gardiner DM, Goff S, Hammond-Kosack KE, Hilburn K, Hua-Van A, Jonkers W, Kazan K, Kodira CD, Koehrsen M, Kumar L, Lee YH, Li L, Manners JM, Miranda-Saavedra D, Mukherjee M, Park G, Park J, Park SY, Proctor RH, Regev A, Ruiz-Roldan MC, Sain D, Sakthikumar S, Sykes S, Schwartz DC, Turgeon BG, Wapinski I, Yoder O, Young S, Zeng Q, Zhou S, Galagan J, Cuomo CA, Kistler HC, Rep M 2010 Comparative genomics reveals mobile pathogenicity chromosomes in *Fusarium*. Nature 464:367–373. doi:10.1038/nature08850. 20237561 PMC3048781

[14] Galagan JE, Calvo SE, Borkovich KA, Selker EU, Read ND, Jaffe D, FitzHugh W, Ma LJ, Smirnov S, Purcell S, Rehman B, Elkins T, Engels R, Wang S, Nielsen CB, Butler J, Endrizzi M, Qui D, Ianakiev P, Bell-Pedersen D, Nelson MA, Werner-Washburne M, Selitrennikoff CP, Kinsey JA, Braun EL, Zelter A, Schulte U, Kothe GO, Jedd G, Mewes W, Staben C, Marcotte E, Greenberg D, Roy A, Foley K, Naylor J, Stange-Thomann N, Barrett R, Gnerre S, Kamal M, Kamvysselis M, Mauceli E, Bielke C, Rudd S, Frishman D, Krystofova S, Rasmussen C, Metzenberg RL, Perkins DD, Kroken S, Cogoni C, Macino G, Catcheside D, Li W, Pratt RJ, Osmani SA, DeSouza CP, Glass L, Orbach MJ, Berglund JA, Voelker R, Yarden O, Plamann M, Seiler S, Dunlap J, Radford A, Aramayo R, Natvig DO, Alex LA, Mannhaupt G, Ebbole DJ, Freitag M, Paulsen I, Sachs MS, Lander ES, Nusbaum C, Birren B 2003 The genome sequence of the filamentous fungus *Neurospora crassa*. Nature 422:859–868. doi:10.1038/nature01554. 12712197

[15] Baroncelli R, Sreenivasaprasad S, Sukno SA, Thon MR, Holub E 2014 Draft genome sequence of *Colletotrichum acutatum sensu lato* (*Colletotrichum fioriniae*). Genome Announc 2(2):e00112-14. doi:10.1128/genomeA.00112-14. 24723700 PMC3983289

[16] Baroncelli R, Sanz-Martín JM, Rech GE, Sukno SA, Thon MR 2014 Draft genome sequence of *Colletotrichum sublineola*, a destructive pathogen of cultivated sorghum. Genome Announc 2(3):e00540-14. doi:10.1128/genomeA.00540-14. 24926053 PMC4056296

[17] Dean RA, Talbot NJ, Ebbole DJ, Farman ML, Mitchell TK, Orbach MJ, Thon M, Kulkarni R, Xu JR, Pan H, Read ND, Lee YH, Carbone I, Brown D, Oh YY, Donofrio N, Jeong JS, Soanes DM, Djonovic S, Kolomiets E, Rehmeyer C, Li W, Harding M, Kim S, Lebrun MH, Bohnert H, Coughlan S, Butler J, Calvo S, Ma LJ, Nicol R, Purcell S, Nusbaum C, Galagan JE, Birren BW 2005 The genome sequence of the rice blast fungus *Magnaporthe grisea*. Nature 434:980–986. doi:10.1038/nature03449. 15846337

[18] Karlsson M, Durling MB, Choi J, Kosawang C, Lackner G, Tzelepis GD, Nygren K, Dubey MK, Kamou N, Levasseur A, Zapparata A, Wang J, Amby DB, Jensen B, Sarrocco S, Panteris E, Lagopodi AL, Pöggeler S, Vannacci G, Collinge DB, Hoffmeister D, Henrissat B, Lee YH, Jensen DF 2015 Insights on the evolution of mycoparasitism from the genome of *Clonostachys rosea*. Genome Biol Evol 7:465–480. doi:10.1093/gbe/evu292. 25575496 PMC4350171

[19] Klosterman SJ, Subbarao KV, Kang S, Veronese P, Gold SE, Thomma BP, Chen Z, Henrissat B, Lee YH, Park J, Garcia-Pedrajas MD, Barbara DJ, Anchieta A, de Jonge R, Santhanam P, Maruthachalam K, Atallah Z, Amyotte SG, Paz Z, Inderbitzin P, Hayes RJ, Heiman DI, Young S, Zeng Q, Engels R, Galagan J, Cuomo CA, Dobinson KF, Ma LJ 2011 Comparative genomics yields insights into niche adaptation of plant vascular wilt pathogens. PLoS Pathog 7:e1002137. doi:10.1371/journal.ppat.1002137. 21829347 PMC3145793

